# Combining quantitative and qualitative breast density measures to assess breast cancer risk

**DOI:** 10.1186/s13058-017-0887-5

**Published:** 2017-08-22

**Authors:** Karla Kerlikowske, Lin Ma, Christopher G. Scott, Amir P. Mahmoudzadeh, Matthew R. Jensen, Brian L. Sprague, Louise M. Henderson, V. Shane Pankratz, Steven R. Cummings, Diana L. Miglioretti, Celine M. Vachon, John A. Shepherd

**Affiliations:** 1 Department of Epidemiology and Biostatistics, University of California, San Francisco, CA USA; 2 General Internal Medicine Section, San Francisco Veterans Affairs Medical Center, 111A1, 4150 Clement Street, San Francisco, CA 94121 USA; 3 Department of Medicine, University of California, San Francisco, CA USA; 4 Division of Biomedical Statistics and Informatics, Department of Health Sciences Research, Mayo Clinic College of Medicine, Rochester, MN USA; 5 Department of Radiology, University of California, San Francisco, CA USA; 6 Department of Surgery, University of Vermont, Burlington, VT USA; 7 Department of Radiology, School of Medicine, University of North Carolina, Chapel Hill, NC USA; 8 Department of Internal Medicine, University of New Mexico, Albuquerque, NM USA; 9 San Francisco Coordinating Center, California Pacific Medical Center Research Institute, San Francisco, CA USA; 10 Department of Public Health Sciences, University of California, Davis, CA USA; 11 Group Health Research Institute, Group Health Cooperative, Seattle, WA USA

**Keywords:** Breast density, Breast cancer risk, Dense volume

## Abstract

**Background:**

Accurately identifying women with dense breasts (Breast Imaging Reporting and Data System [BI-RADS] heterogeneously or extremely dense) who are at high breast cancer risk will facilitate discussions of supplemental imaging and primary prevention. We examined the independent contribution of dense breast volume and BI-RADS breast density to predict invasive breast cancer and whether dense breast volume combined with Breast Cancer Surveillance Consortium (BCSC) risk model factors (age, race/ethnicity, family history of breast cancer, history of breast biopsy, and BI-RADS breast density) improves identifying women with dense breasts at high breast cancer risk.

**Methods:**

We conducted a case-control study of 1720 women with invasive cancer and 3686 control subjects. We calculated ORs and 95% CIs for the effect of BI-RADS breast density and Volpara™ automated dense breast volume on invasive cancer risk, adjusting for other BCSC risk model factors plus body mass index (BMI), and we compared C-statistics between models. We calculated BCSC 5-year breast cancer risk, incorporating the adjusted ORs associated with dense breast volume.

**Results:**

Compared with women with BI-RADS scattered fibroglandular densities and second-quartile dense breast volume, women with BI-RADS extremely dense breasts and third- or fourth-quartile dense breast volume (75% of women with extremely dense breasts) had high breast cancer risk (OR 2.87, 95% CI 1.84–4.47, and OR 2.56, 95% CI 1.87–3.52, respectively), whereas women with extremely dense breasts and first- or second-quartile dense breast volume were not at significantly increased breast cancer risk (OR 1.53, 95% CI 0.75–3.09, and OR 1.50, 95% CI 0.82–2.73, respectively). Adding continuous dense breast volume to a model with BCSC risk model factors and BMI increased discriminatory accuracy compared with a model with only BCSC risk model factors (C-statistic 0.639, 95% CI 0.623–0.654, vs. C-statistic 0.614, 95% CI 0.598–0.630, respectively; *P* < 0.001). Women with dense breasts and fourth-quartile dense breast volume had a BCSC 5-year risk of 2.5%, whereas women with dense breasts and first-quartile dense breast volume had a 5-year risk ≤ 1.8%.

**Conclusions:**

Risk models with automated dense breast volume combined with BI-RADS breast density may better identify women with dense breasts at high breast cancer risk than risk models with either measure alone.

## Background

Many studies using both qualitative and quantitative breast density measures have found women with high breast density (greater amount of breast and connective tissue compared with fat) are at increased breast cancer risk [1]. The Breast Imaging Reporting and Data System (BI-RADS) breast density categories estimated subjectively by radiologists [2] is the standard for reporting breast density in clinical practice in the United States. Quantitative breast density measures are now available with commercial (Quantra^TM^, Hologic, Inc., Marlborough, MA, USA; and Volpara^TM^, Volpara Solutions/Matakina Technology, Wellington, New Zealand) and publicly available (Laboratory for Individualized Breast Radiodensity Assessment [LIBRA]) software that can be used in clinical practice. Breast cancer associations appear to be similar with qualitative and quantitative measures examined [3–5].

BI-RADS breast density is estimated visually and reflects density quantity, distribution, and parenchymal pattern, whereas quantitative measures algorithmically assess absolute dense breast volume. The correlation of clinical BI-RADS density with quantitative dense breast volume (Quantra^TM^, Volpara^TM^) is modest [6–8]. Discordant associations between BI-RADS density and dense breast volume have been reported for black and Asian women. Asian women have a higher proportion of dense breasts (BI-RADS heterogeneously or extremely dense) than white women, but dense breast volume is lower in Asian women [4]. Conversely, black and white women have a similar proportion with dense breasts, but dense breast volume is higher in black than in white women [8]. This suggests clinical BI-RADS density and quantitative density measures may be measuring different aspects of breast density and possibly breast cancer risk.

About 50% of women with dense breasts are at low to average breast cancer risk (5-year risk ≤ 1.67%) [9]. Accurately identifying women with dense breasts who are at high breast cancer risk (5-year risk ≥ 2.5%) will facilitate discussions of supplemental imaging and primary prevention. Combining a qualitative or quantitative breast density measure with clinical risk factors estimates a woman’s breast cancer risk more accurately than does either measure alone [3, 10–12]. No breast cancer risk prediction models have combined clinical risk factors and qualitative and quantitative measures of breast density. An advantage of incorporating dense breast volume into risk prediction models is that it is independent of clinical risk factors and only weakly confounded by body mass index (BMI) [4].

We evaluated the independent contribution and improved discriminatory accuracy of adding dense breast volume to the Breast Cancer Surveillance Consortium (BCSC) risk prediction model [3], which includes age, race/ethnicity, first-degree family history of breast cancer, history of breast biopsy, and BI-RADS breast density with additional adjustment for BMI to identify women with dense breasts who are at high breast cancer risk.

## Methods

### Study sample

Study participants were in one of three case-control studies nested within large prospective breast imaging cohorts. The San Francisco Mammography Registry (SFMR) and Vermont Breast Cancer Surveillance System (VBCSS) participate in the National Cancer Institute-funded BCSC (http://www.bcsc-research.org/) [13]. Both registries obtain annual institutional review board approval and passive permission for data collection and enrollment of participants, as well as data linkages for research purposes, and both received a Federal Certificate of Confidentiality that protects the identities of research participants. For the Mayo Clinic screening cohort, a waiver of informed consent and Health Insurance Portability and Accountability Act authorization from the participants was approved by the institutional review board. Only individuals who had not refused permission to use their medical records for research (according to Minnesota Research Authorization Statute) were included from the Mayo Clinic cohort [14].

The SFMR obtained “for processing” digital screening examinations done with Selenia machines (Hologic Inc.) at four facilities since 2006, and the VBCSS obtained them from one facility for screenings done since 2007, which serve as the underlying screening mammography cohort for each registry. Incident invasive breast cancers reported to the California Cancer Registry (*n* = 1052) or the Vermont Cancer Registry and pathology databases (*n* = 288) from January 2007 through November 2012 with a raw digital screening examination at least 6 months prior to diagnosis with no upper time limit were included. Two control subjects (*n* = 2678) without prior breast cancer were matched to each case on age within 5 years, race, date of screening examination within 1 year, mammography machine, and facility. The Mayo Clinic cohort obtained “for processing” digital images from Selenia machines from April 2008 through December 2013 from women in the tristate region of Minnesota, Iowa, and Wisconsin. Incident invasive breast cancers from this region reported to the Mayo Clinic tumor registry through December 2013 (*n* = 380) were included. Approximately three control subjects (*n* = 1008) without prior breast cancer were matched to each case on age within 5 years, race, state of residence, date of screening examination within 1 year, and mammography machine. We ensured that each control subject had at least one normal screening mammogram on or after the corresponding case’s diagnosis date. Our total sample consisted of 1720 invasive cases and 3686 control subjects.

### Measurement of risk factors

Information on age, first-degree family history of breast cancer, race/ethnicity, prior breast biopsy history, height, weight, and age at first live birth were obtained from self-report (SFMR and VBCSS) at the time of mammography and from self-report or medical record review (height and weight) for the Mayo Clinic cohort. BMI was calculated by dividing weight in kilograms by height in square meters. Race/ethnicity was coded using the expanded definition currently used in the Surveillance, Epidemiology, and End Results program and U.S. vital statistics (non-Hispanic White, non-Hispanic black, Asian/Pacific Islander, Native American/Alaskan Native, Hispanic, other/mixed race). Age at first live birth was dichotomized as nulliparous or age ≥ 30 years for first live birth compared with age at first birth < 30 years.

### BI-RADS breast density and dense breast volume

Practicing radiologists classified breast density as part of routine clinical practice at the time of mammography interpretation using the BI-RADS® density categories [2]: a = almost entirely fat, b = scattered fibroglandular densities, c = heterogeneously dense, and d = extremely dense. Volpara™ version 1.5.0, which is the most common automated 3D density measurement tool used in clinical practice and research settings, is a fully automated method for assessing volumetric breast density that uses the measured breast thickness and X-ray attenuations in the “for processing” image to create estimates of dense and nondense tissue volume for each pixel. Summing the dense pixel volumes provides total dense breast volume. Volpara uses proprietary algorithms to calculate breast thickness and determine dense tissue volume [15] by averaging measures of each breast. For this study, we used the dense breast volume output from the vendor-specific software per woman, incorporating all four views (craniocaudal and mediolateral oblique of both breasts) of raw digital images as done in the clinical setting.

### Statistical methods

We compared frequency distributions of demographics and risk factors between cases and control subjects. Dense breast volume quartiles were defined using control subjects from all sites. Within each BI-RADS category, the proportion of cases and control subjects within each dense breast volume quartile was calculated. Spearman’s correlation coefficient was calculated to assess the association of BI-RADS density categories with continuous dense breast volume.

Conditional logistic regression stratified on matched set was used to examine the effects of BI-RADS density and dense breast volume on invasive breast cancer risk. Associations were summarized with ORs and 95% CIs, and with AUROC or C-statistics, which accounted for the matched study design. Differences in C-statistics between models were tested using the SEs estimated from 5000 bootstrap samples. Models were adjusted for age, race/ethnicity, first-degree family history of breast cancer, history of benign breast biopsy, and BMI (continuous). We used the second BI-RADS category as a reference to allow for estimations of risk at the lowest and highest categories and because it is the most prevalent category. Study heterogeneity was evaluated by including an interaction term between study site and breast density measurement. Initially, we fit a model evaluating the association among all possible combinations of BI-RADS density and quartiles of dense breast volume in reference to women with scattered fibroglandular density and second-quartile dense breast volume. Differences in breast cancer association among combinations of BI-RADS density and quartiles of dense breast volume were evaluated by including a multiplicative interaction term. Then, separate models were evaluated that included BCSC clinical risk factors, with and without BMI, plus one or more measure(s) of breast density: BI-RADS density, continuous dense breast volume, and BI-RADS density plus continuous dense breast volume. Continuous dense breast volume was log-transformed and divided by its SD.

To examine the absolute risk associated with quartiles of dense breast volume, we incorporated the adjusted ORs associated with dense breast volume into the BCSC model. ORs for each quartile from the fully adjusted model were standardized for attributable fraction so that they measured increased or decreased risk relative to average risk. We calculated the estimated 5-year absolute risk from both the original model and the updated model for each subject and summarized these absolute risks across BI-RADS and dense breast volume categories. For those with heterogeneously or extremely dense breasts, the proportion of control subjects with risks ≥ 2.5% were calculated for each model. These proportions were then used to calculate the net gain in reclassification for the updated risk model including dense breast volume.

Analyses were performed using SAS version 9.4 software (SAS Institute, Cary, NC, USA). Two-sided statistical tests were used, and *P* values < 0.05 were considered to be statistically significant.

## Results

We compared 1720 women with invasive breast cancer with 3686 matched women without breast cancer with breast density measured, on average, 2.4 years (range 6 months to 6 years) before cancer diagnosis. Women with invasive cancer were more likely to be nulliparous or ≥ 30 years of age at first live birth, to have a family history of breast cancer, to have heterogeneously or extremely dense breasts, and to have higher mean dense breast volume (Table 1). A wide distribution of dense breast volume was observed within each BI-RADS density category (Table 2). Surprisingly, about one-third (30.5%) of control subjects with almost entirely fat breasts had first-quartile dense breast volume (≤35.9 ml), and about half (54.1%) with extremely dense breasts had fourth-quartile (>70.0 ml) dense breast volume. The correlation coefficient between continuous dense breast volume and BI-RADS density was *r* = 0.38 (95% CI 0.34–0.42) for cases and *r* = 0.31 (95% CI 0.29–0.34) for control subjects.

**Table 1:** Characteristics of study sample

	Invasive breast cancer cases (*n* = 1720)	Matched control subjects (*n* = 3686)
Age, years, mean (SD)	59.5 (12.1)	59.6 (12.0)
Body mass index, kg/m^2^, mean (SD)	26.3 (5.7)	26.2 (5.7)
Dense breast volume,^a^ ml, median (IQR)	57.8 (41.0–82.5)	50.7 (36.8–70.2)
Age at first live birth, *n* (%)		
< 30 years	856 (50.1%)	2085 (56.6%)
None or ≥ 30 years	852 (49.9%)	1600 (43.4%)
Family history of breast cancer,^b^ *n* (%)		
No	1218 (71.2%)	2991 (81.2%)
Yes	493 (28.8%)	693 (18.8%)
History of breast biopsy, *n* (%)		
No	1304 (76.4%)	3040 (82.6%)
Yes	402 (23.6%)	641 (17.4%)
Race/ethnicity, *n* (%)		
White	1380 (80.3%)	3012 (81.7%)
Asian	218 (12.7%)	437 (11.9%)
Black	34 (2.0%)	67 (1.8%)
Hispanic	37 (2.2%)	101 (2.7%)
Other	50 (2.9%)	69 (1.9%)
BI-RADS breast density,^c^ *n* (%)		
Almost entirely fat (a)	233 (13.5%)	731 (19.8%)
Scattered fibroglandular densities (b)	668 (38.8%)	1557 (42.2%)
Heterogeneously dense (c)	600 (34.9%)	1115 (30.2%)
Extremely dense (d)	219 (12.7%)	283 (7.7%)

*BI-RADS* Breast Imaging Reporting and Data System

Data are presented as number (percent) missing for cases and control subjects for age at first birth (13 [0.2%]), family history of breast cancer (11 [0.2%]), history of breast biopsy (19 [0.4%]), and race/ethnicity (3 [0.1%])

^a^Measured with Volpara software

^b^Mother, sister, or daughter with breast cancer

^c^BI-RADS breast density: a = almost entirely fat, b = scattered fibroglandular densities, c = heterogeneously dense, d = extremely dense

**Table 2:** Frequency distribution of volumetric density within Breast Imaging Reporting and Data System breast density categories

Dense breast volume,^b^ ml	Control subjects				Cases			
	BI-RADS breast density^a^				BI-RADS breast density^a^			
	a *n* = 731 %	b *n* = 1557 %	c *n* = 1115 %	d *n* = 283 %	a *n* = 233 %	b *n* = 668 %	c *n* = 600 %	d *n* = 219 %
Quartile 1: ≤ 35.9	30.5	28.0	15.2	9.9	26.2	24.4	9.7	5.9
Quartile 2: 36.0–50.0	32.1	28.5	19.3	15.2	30.0	27.7	17.3	9.1
Quartile 3: 50.1–70.0	27.5	26.3	26.3	20.8	29.6	26.9	25.7	23.7
Quartile 4: 70.1+	9.8	17.2	39.3	54.1	14.2	21.0	47.3	61.2

*BI-RADS* Breast Imaging Reporting and Data System

^a^BI-RADS breast density: a = almost entirely fat, b = scattered fibroglandular densities, c = heterogeneously dense, d = extremely dense

^b^Measured with Volpara software

### Quartiles of dense breast volume and BI-RADS density associations with breast cancer risk

In multivariable models that included clinical risk factors in the BCSC risk model with additional adjustment for BMI, compared with women with BI-RADS scattered fibroglandular density and second-quartile dense breast volume, women with BI-RADS extremely dense breasts and third or fourth-quartile dense breast volume (75% of women with extremely dense breasts) had high breast cancer risk (OR 2.87, 95% CI 1.84–4.47, and OR 2.56, 95% CI 1.87–3.52, respectively), whereas women with extremely dense breasts and first- or second-quartile dense breast volume were not at significantly increased breast cancer risk (OR 1.53, 95% CI 0.75–3.09, and OR 1.50, 95% CI 0.82–2.73, respectively) (Table 3). Women with BI-RADS almost entirely fat breasts and first- or second-quartile dense breast volume (63% of women with fatty breasts) had the lowest breast cancer risk (OR 0.63, 95% CI 0.45–0.89, and OR 0.60, 95% CI 0.43–0.84, respectively). There was no significant interaction between BI-RADS density and dense breast volume associations with breast cancer (*P* = 0.75).

**Table 3:** Breast cancer risk associated with Breast Imaging Reporting and Data System breast density cross-classified with dense breast volume

Dense breast volume,^b^ ml	BI-RADS breast density a^a^	BI-RADS breast density b^a^	BI-RADS breast density c^a^	BI-RADS breast density d^a^	Overall
	OR (95% CI)^c^	OR (95% CI)^c^	OR (95% CI)^c^	OR (95% CI)^c^	OR (95% CI)^c^
Quartile 1: ≤ 35.9	**0.63** (0.45–0.89)	0.94 (0.72–1.21)	1.03 (0.71–1.49)	1.53 (0.75–3.09)	0.85 (0.71–1.03)
Quartile 2: 36.0–50.0	**0.60** (0.43–0.84)	1.00 (reference)	1.25 (0.92–1.69)	1.50 (0.82–2.73)	1.00 (reference)
Quartile 3: 50.1–70.0	**0.70** (0.49–0.99)	0.98 (0.76–1.27)	**1.43** (1.09–1.89)	**2.87** (1.84–4.47)	**1.19 (1.00–1.42)**
Quartile 4: 70.1+	0.87 (0.54–1.41)	1.15 (0.87–1.53)	**1.67** (1.31–2.12)	**2.56** (1.87–3.52)	**1.62 (1.36–1.92)**

*BI-RADS* Breast Imaging Reporting and Data System

^a^BI-RADS breast density: a = almost entirely fat, b = scattered fibroglandular densities, c = heterogeneously dense, d = extremely dense

^b^Measured with Volpara software

^c^Adjusted for age, body mass index, family history of breast cancer, history of breast biopsy, and race/ethnicity. Statistically significant results are shown in boldface type

### Continuous dense breast volume and BI-RADS density and breast cancer risk associations

In multivariable models that included risk factors in the BCSC risk model with additional adjustment for BMI, women with BI-RADS extremely dense breasts were at 2.45 times higher risk than women with scattered fibroglandular densities (Table 4). In a separate model, continuous dense breast volume increased risk 33% per 1-SD increase in dense breast volume. When combining continuous dense breast volume and BI-RADS density in the same multivariable model, associations with breast cancer were attenuated, but both density variables remained statistically significant (Table 4). There was no evidence of study heterogeneity for models with BI-RADS density or dense breast volume assessed by quartile or continuously (*P* = 0.95, *P* = 0.71, and *P* = 0.41, respectively).

**Table 4:** Multivariable models with Breast Imaging Reporting and Data System breast density, dense breast volume, or both, with and without adjustment for body mass index

	BI-RADS breast density	Continuous dense breast volume	BI-RADS breast density + continuous dense breast volume	BI-RADS breast density adjusted for BMI	Continuous dense breast volume adjusted for BMI	BI-RADS breast density + continuous dense breast volume adjusted for BMI
	OR^a^ (95% CI)	OR^a^ (95% CI)	OR^a^ (95% CI)	OR^b^ (95% CI)	OR^b^ (95% CI)	OR^b^ (95% CI)
BI-RADS breast density						
Almost entirely fat = a	**0.74** (0.62–0.89)		**0.77** (0.64–0.92)	**0.65** (0.54–0.78)		**0.69** (0.57–0.84)
Scattered fibroglandular densities = b	1.00 (reference)		1.00 (reference)	1.00 (reference)		1.00 (reference)
Heterogeneously dense = c	**1.34** (1.16–1.55)		**1.17** (1.01–1.36)	**1.45** (1.25–1.68)		**1.26** (1.07–1.48)
Extremely dense = d	**2.01** (1.61–2.51)		**1.63** (1.30–2.05)	**2.45** (1.93–3.09)		**1.93** (1.50–2.48)
Dense breast volume, ml, per SD^c^		**1.34** (1.26–1.42)	**1.26** (1.18–1.35)		**1.33** (1.25–1.42)	**1.20** (1.11–1.29)
C-statistic^d^	0.614 (0.598–0.630)	0.623 (0.607–0.639)	0.627 (0.611–0.642)	0.634 (0.618–0.649)	0.630 (0.614–0.645)	0.639 (0.623–0.654)

*BI-RADS* Breast Imaging Reporting and Data System, *BMI* Body mass index

^a^Adjusted for age, race/ethnicity, history of breast biopsy, family history of breast cancer. Statistically significant results are shown in boldface type

^b^Adjusted for age, race/ethnicity, history of breast biopsy, family history of breast cancer, BMI. Statistically significant results are shown in boldface type

^c^Measured with Volpara software, log-transformed

^d^C-statistic represents the AUROC

### Model discrimination

Adding continuous dense breast volume to a model with BCSC risk model factors showed improved discrimination of case status (C-statistic 0.627, 95% CI 0.611–0.642, vs. C-statistic 0.614, 95% CI 0.598–0.630, respectively; *P* = 0.02) (Table 4). Adding continuous dense breast volume to a model with BCSC risk model factors and BMI further increased discriminatory accuracy compared with a model with only BCSC risk model factors (C-statistic 0.639, 95% CI 0.623–0.654, vs. C-statistic 0.614, 95% CI 0.598–0.630, respectively; *P* < 0.001). Adding quartiles of dense breast volume to a model with BCSC risk model factors and BMI had less discrimination than a model with continuous dense breast volume (C-statistic 0.629, 95% CI 0.614–0.645) (model results not shown).

### Absolute breast cancer risk combining BI-RADS density and dense breast volume

The mean 5-year breast cancer risk for women with BI-RADS heterogeneously or extremely dense breasts is 2.0%. The 5-year risk is higher when considering BI-RADS density and dense breast volume (Table 5). For example, women with BI-RADS heterogeneously or extremely dense breasts and fourth-quartile dense breast volume had a 5-year risk of 2.5%. Overall, the proportion of women with heterogeneously or extremely dense breasts with 5-year risk ≥ 2.5% increased from 20.3% to 30.5% when combined with a dense breast volume measure. The proportion of cancers with heterogeneously dense breasts and 5-year risk of ≥ 2.5% increased from 24.3% to 37.9% when taking into account a continuous dense breast volume measure, whereas control subjects increased from 19.2% to 29.8%, for a net gain of 3.2% of high-risk women identified as having cancer. For women with extremely dense breasts, the proportion of breast cancers increased from 29.2% to 43.1% when taking into account a continuous dense breast volume measure, and that of control subjects increased from 24.8% to 34.0%, for a net gain of 4.7% of high-risk women identified as having cancer. Dense breast volume did not change the predicted risk significantly for women with BI-RADS almost entirely fat or scattered fibroglandular breast density.

**Table 5:** Breast Cancer Surveillance Consortium 5-year breast cancer risk by Breast Imaging Reporting and Data System breast density with the addition of dense breast volume

	BI-RADS breast density a^a^	BI-RADS breast density b^a^	BI-RADS breast density c^a^	BI-RADS breast density d^a^
	5-year risk, mean (SD)	5-year risk, mean (SD)	5-year risk, mean (SD)	5-year risk, mean (SD)
Dense breast volume,^b^ ml				
Quartile 1: ≤ 35.9	0.9 (0.5)	1.6 (0.9)	1.8 (1.1)	1.5 (0.8)
Quartile 2: 36.0–50.0	1.1 (0.6)	1.7 (0.9)	2.1 (1.3)	2.3 (1.7)
Quartile 3: 50.1–70.0	1.0 (0.7)	1.8 (1.1)	2.2 (1.3)	2.4 (1.6)
Quartile 4: 70.1+	1.2 (0.7)	1.9 (1.2)	2.5 (1.5)	2.5 (1.6)
Mean BCSC 5-year risk	1.0 (0.6)	1.7 (1.0)	2.0 (1.2)	2.0 (1.4)

*BCSC* Breast Cancer Surveillance Consortium, *BI-RADS* Breast Imaging Reporting and Data System

^a^BI-RADS breast density: a = almost entirely fat, b = scattered fibroglandular densities, c = heterogeneously dense, d = extremely dense

^b^Measured with Volpara software

## Discussion

We found that adding a continuous measure of dense breast volume to risk prediction models which include BI-RADS breast density and clinical risk factors improves discriminatory accuracy, in particular for women with heterogeneously or extremely dense breasts. Among women aged 40–74 years, an estimated 43.3% of women have dense breasts [16], but not all women with dense breasts are at increased risk of breast cancer [9]. Adding a volumetric breast density measure may improve discrimination of women with dense breasts who are considering supplemental screening or primary prevention, given the wide variation in BI-RADS density assessment across radiologists [17].

There are no evidence-based consensus guidelines for screening women with dense breasts. A 2013 Cochrane review and the evidence review for the 2015 U.S Preventive Services Task Force recommendations concluded that no data currently exist to provide evidence for or against the use of supplemental screening ultrasonography in women with dense breasts, and they challenged investigators to provide such evidence [18, 19]. The 2010 guidelines of the Society of Breast Imaging and the American College of Radiology recommend that ultrasound be considered as supplemental screening for women at high risk and in women with dense breasts [20]. Fifty percent of women with dense breasts have low breast cancer risk (<1.67% 5-year risk) [9]. Thus, combinations of breast cancer risk factors and qualitative and quantitative measures of breast density could be used to better identify women with dense breasts at high breast cancer risk who may benefit from a more intense screening strategy.

The variability in the patterns and amounts of dense tissue portrayed on the mammogram has been characterized by qualitative measures. The Wolfe scale qualitatively assesses breast density by classifying images into one of four patterns (N1 [fatty], P1, P2 [areas of increasing ductal prominence], and DY [significant densities or dysplasia]) on the basis of quantity and distribution of breast density [21]. The BI-RADS density qualitative assessment is similar to the Wolfe scale in that it incorporates the quantity, distribution, and texture of breast density. New automated commercial technologies are available to quantify breast density volume using methods such as Volpara^TM^ and Quantra^TM^. Texture features or parenchymal complexity has been evaluated on film-screen mammography examinations and shown to be independent of percentage breast density and relative amounts of fibroglandular tissue [22, 23]. Winkel et al. [22] showed that the discriminatory accuracy was highest when a combination of three methods of assessing relative amounts of density and texture on film-screen mammography examinations were combined. Automated evaluation of textural features or parenchymal complexity of breast density is under investigation for digital images, with researchers in several studies reporting features as independent predictors of breast cancer risk [24–26]. Our results extend the literature by showing that an automated quantitative breast density measure (dense breast volume) available in clinical practice combined with a qualitative measure that incorporates the quantity, distribution, and texture of breast density (BI-RADS breast density) improves the classification of women with dense breasts at high breast cancer risk.

Among women with dense breasts, 24% are at high risk of an interval cancer (>1 per 1000 screening examinations) and warrant discussions of supplemental imaging [9]. Interval cancer rates are highest among women with extremely or heterogeneously dense breasts and BCSC 5-year breast cancer risk of 2.5% or higher (21% of women with dense breasts) [9]. If continuous dense breast volume was assessed in combination with BCSC risk, 30.5% of women with dense breasts would have a 5-year risk of 2.5% or higher and qualify for discussions of supplemental imaging or hormone therapy for primary prevention [27]. Moreover, continuous dense breast volume assessed in combination with BCSC model risk factors increases the identification of women with dense breasts at high breast cancer risk to a greater extent than measuring single-nucleotide polymorphisms [28]. Future studies should confirm whether interval cancer rates are higher among women with BI-RADS dense breasts and high dense breast volume than BI-RADS density alone.

This is the largest study to date involving an examination of the combination of an automated quantitative measure of dense breast volume and BI-RADS breast density on digital mammography examinations and their independent contribution to breast cancer risk. We examined Volpara’s automated dense breast volume measure. Other commercially (Quantra^TM^) and publicly (LIBRA) [29] available automated breast density software could be tested to verify our results. We used clinical BI-RADS density assessments when the definitions from the BI-RADS fourth edition were available in clinical practice. Studies using data collected since the release of the BI-RADS fifth edition, whose categories attempt to put more emphasis on the masking effect of dense tissue, should be assessed to ensure our results are robust. In studies for interrater and intrarater reliability of the BI-RADS categories, investigators have reported moderate to substantial agreement [30–32]. Thus, misclassification of BI-RADS categories may have influenced our results, such that some of the differences we observed could result in an under- or overestimation of associations [33]. Our population was predominantly white and Asian; studies should be repeated with black and Hispanic women to ensure generalizability of results across all racial/ethnic groups. Last, our study included women undergoing digital mammography, which is the primary modality used in the United States. Breast tomosynthesis is an emerging breast imaging modality that is being used for breast screening in 40% of certified U.S. facilities as of August 1, 2017 [34]; as such, the independent contribution of qualitative and quantitative density measures to breast cancer risk for this modality needs to be established.

## Conclusions

We found that the combination of automated quantitative and qualitative clinical assessments of breast density can better identify women with dense breasts at high breast cancer risk than either measure alone. In future studies, researchers should assess automated dense breast volume measures in combination with automated parenchymal complexity features, as well as qualitative breast density measures, because both may measure different aspects of breast density than quantitative measures when assessing breast cancer risk and screening outcomes [35].

## Abbreviations

BCSC: Breast Cancer Surveillance Consortium
BI-RADS: Breast Imaging Reporting and Data System
BMI: Body mass index
LIBRA: Laboratory for Individualized Breast Radiodensity Assessment
NCI: National Cancer Institute
SFMR: San Francisco Mammography Registry
VBCSS: Vermont Breast Cancer Surveillance System
